# Rescue bare-metal stent placement restoring multilevel perfusion in acute type B aortic dissection: a case report

**DOI:** 10.3389/fcvm.2025.1695877

**Published:** 2026-01-06

**Authors:** Ken Nakamura, Shusuke Arai, Cholsu Kim, Hideaki Uchino

**Affiliations:** Division of Cardiovascular Surgery, Nihonkai General Hospital, Sakata, Japan

**Keywords:** type B acute aortic dissection, thoracic endovascular aortic repair, lower limb malperfusion, self-expanding stent, extra-anatomic bypass

## Abstract

We report a rare, life-saving endovascular intervention in a patient with acute type B aortic dissection (TBAD) complicated by severe lower limb ischemia due to dynamic obstruction. A 67-year-old woman, previously diagnosed with uncomplicated Stanford type B dissection with a thrombosed false lumen, suddenly developed bilateral leg pain and absent distal pulses on day 12. CT revealed a new entry tear in the descending aorta, leading to false lumen reperfusion and collapse of the true lumen at the abdominal aorta and both common iliac arteries. As appropriate thoracic endovascular aortic repair and endovascular aortic repair devices were not immediately available, emergency repair was performed using four self-expanding bare-metal stents via bilateral femoral access. This achieved prompt true lumen re-expansion and restoration of distal pulses. Post-procedure imaging confirmed improved perfusion. Although extra-anatomical bypass is often considered, complete collapse of the abdominal true lumen made it unlikely to be effective. This case demonstrates that widely available bare-metal stents offer a practical and effective emergency option for restoring distal perfusion when standard devices are not accessible. Importantly, this approach should be regarded as palliative for limb salvage rather than definitive treatment of the entry tear.

## Introduction

Acute aortic dissection is life-threatening, with organ malperfusion significantly increasing morbidity and mortality. About 25% of patients develop acute complications, and visceral malperfusion is linked to a 50%–80% risk (1, 2). This report presents a case of Stanford type B dissection with abdominal aorta and lower limb malperfusion, successfully treated by self-expanding endovascular stenting.

## Case description

A 67-year-old woman with a history of hypertension (BMI 23.9) and a 47-year smoking history (10 cigarettes/day) presented with back pain. There was no relevant family history. On initial hospitalization, her blood pressure was 152/88 mmHg, and Rutherford grade for limb ischemia was 0. Laboratory values included lactate 1.6 mmol/L, creatine kinase (CK) 91 U/L, serum creatinine 0.75 mg/dL, and urine output 100 mL/h. The ankle–brachial index (ABI) was within normal limits.

**Figure 1:**
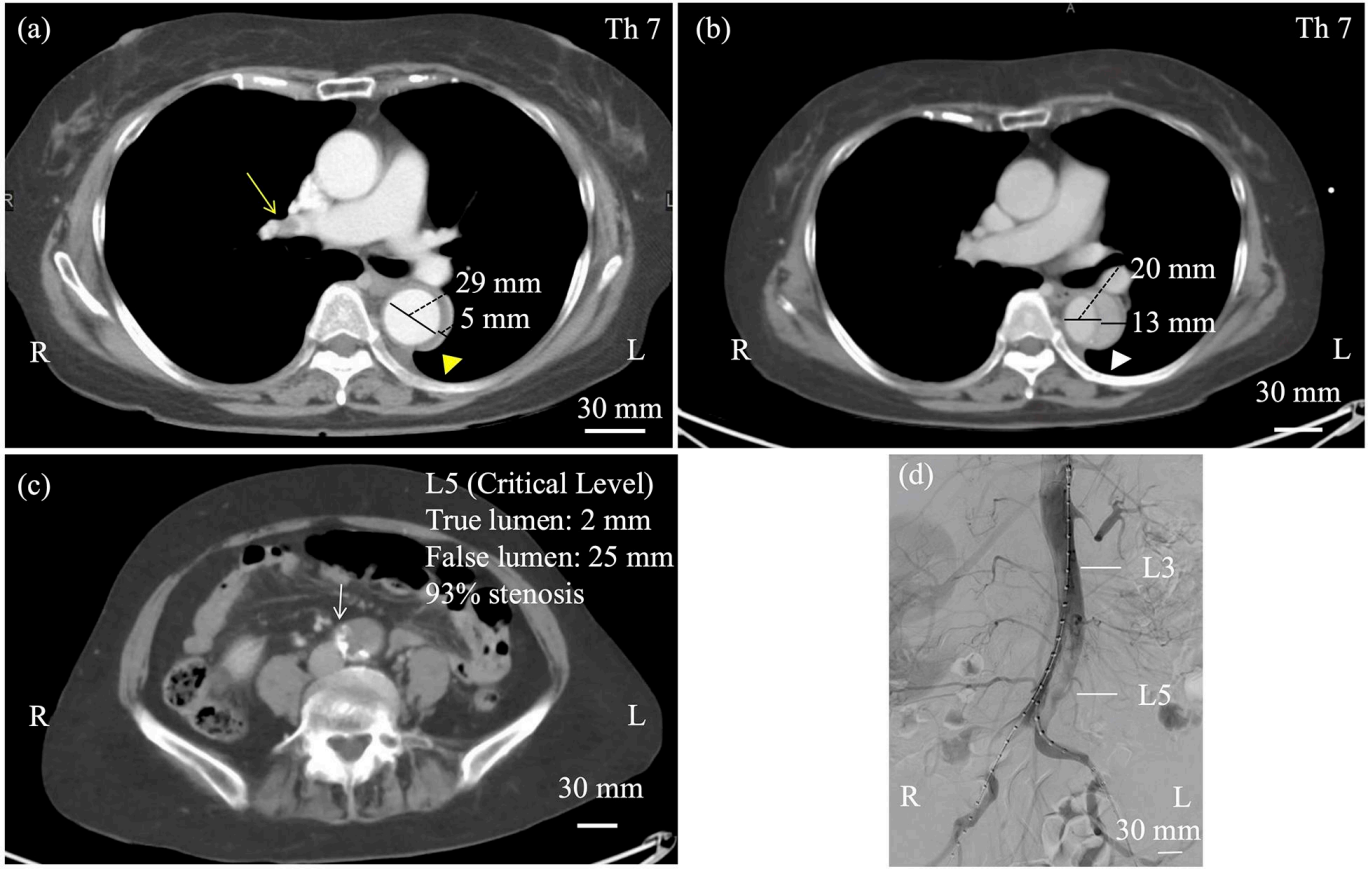
**(a)** Contrast-enhanced computed tomography at day 6 showed right pulmonary artery embolism (yellow arrow) and a thrombosed false lumen in the descending aorta (yellow triangle). The true lumen measured 29 mm, and the false lumen 5 mm at this time. **(b)** At day 12, computed tomographic angiography (CTA) revealed an open false lumen (white triangle) and narrowing of the true lumen (**c**, white arrow), with reduced flow on angiography. At the Th7 level, the true lumen measured 20 mm and the false lumen 13 mm, whereas at the critical L5 level, the true lumen was narrowed to 2 mm and the false lumen expanded to 25 mm. Objective perfusion parameters such as ankle–brachial index (ABI) and renal function were referenced for classification, as shown in the Results section **(d)**.

Contrast-enhanced computed tomography (CT) revealed acute type B aortic dissection (TBAD) with a thrombosed false lumen extending from the descending thoracic to the abdominal aorta. Visceral branches, including the celiac, superior mesenteric, and renal arteries, were perfused via the true lumen. Conservative medical management was initiated. On day 6, although asymptomatic, follow-up CT detected a pulmonary embolism in the right pulmonary artery, and edoxaban 60 mg/day was started (Figure 1a).

On day 12, the patient experienced sudden, severe bilateral leg pain. Femoral pulses were absent bilaterally, while abdominal examination remained unremarkable. Laboratory tests at the time of limb ischemia showed lactate 1.9 mmol/L, CK 1,466 U/L, creatinine 1.19 mg/dL, and urine output 20 mL/h. Lower limb blood pressure was unmeasurable, and Rutherford grade was IIb. Emergency CT demonstrated progression to a patent false lumen (Figure 1b) with the primary entry at the level of Th11, and significant narrowing of the true lumen below the renal arteries, resulting in acute malperfusion of the abdominal aorta and lower extremities (Figure 1c). Digital subtraction angiography confirmed severe stenosis of the infrarenal true lumen (critical levels at L5, 93% stenosis), while major visceral branches remained patent (Figure 1d).

Four hours after symptom onset, bilateral common femoral artery access was obtained using 8 Fr sheaths. After intravenous administration of 7,000 U of heparin, a 0.035-inch guidewire was advanced, and intravascular ultrasound (IVUS) confirmed true lumen positioning. Two self-expanding stents (Epic™ self-expanding nitinol stent, Boston Scientific, USA) were deployed in a kissing configuration above the level of the dynamic obstruction in the abdominal aorta, extending distally from the level of the L3 vertebra. Stent sizes were selected slightly larger than the preoperative true-lumen diameters (8 × 120 mm on the right and 8 × 100 mm on the left). The true-lumen position was confirmed by intravascular ultrasound (IVUS) before deployment. Because this was a dissected aorta with a fragile intimal flap, no balloon post-dilatation was performed to avoid secondary injury. The dynamic obstruction was distal to the visceral branches, so no specific protection of visceral or iliac branches was required. Persistent narrowing in the bilateral common and external iliac arteries was addressed with additional Epic™ stents (8 × 80 mm on the right and 8 × 60 mm on the left). Completion angiography confirmed good perfusion, and heparin was reversed with protamine at the end of the procedure. This procedure is technically straightforward and reproducible using standard endovascular equipment.

**Figure 2:**
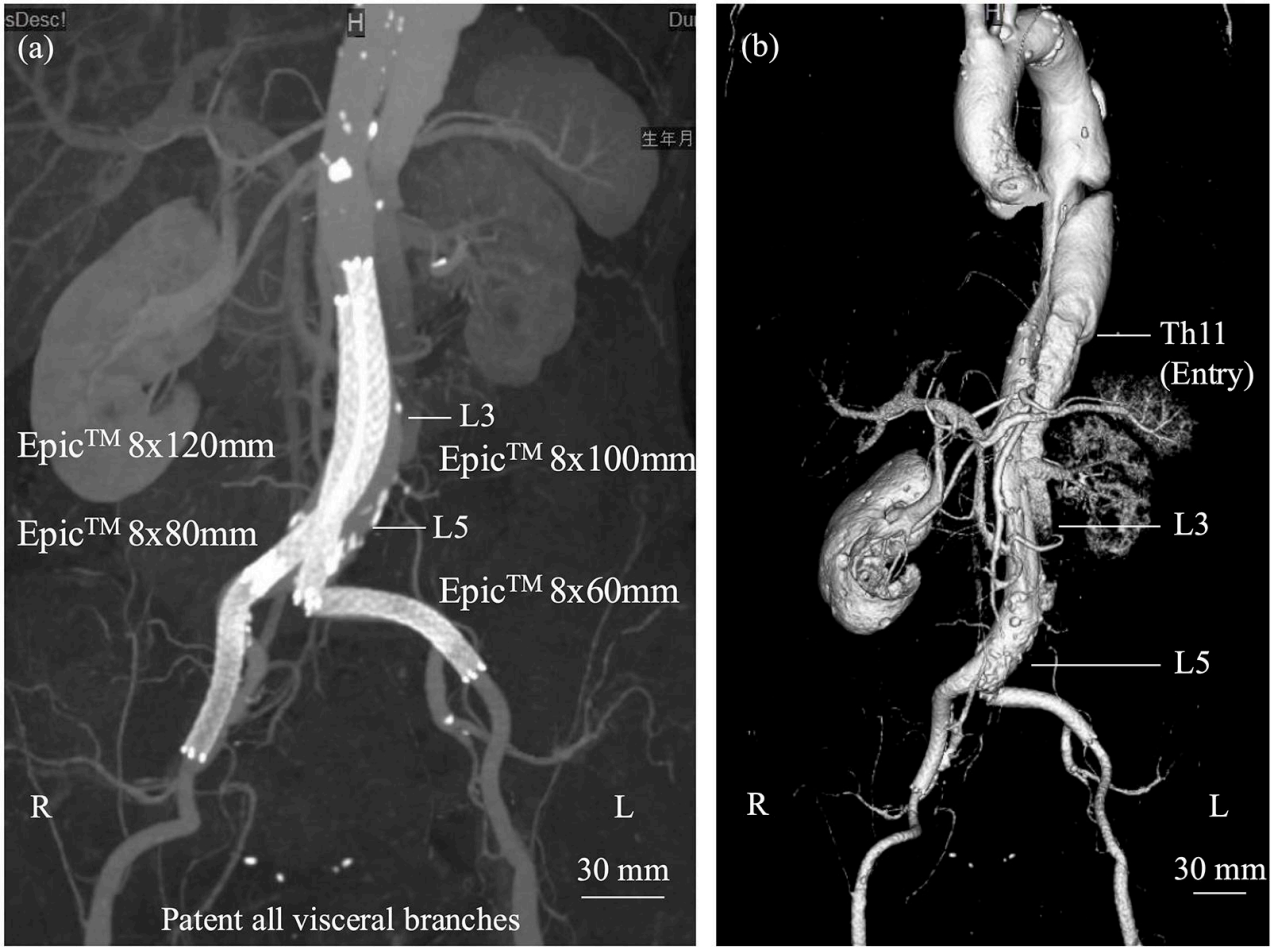
**(a)** Two self-expanding stents were deployed in a kissing configuration within the abdominal aorta, each extending into the respective iliac artery and relined with additional stents. **(b)** Post-discharge computed tomography demonstrated preservation of the true lumen from the abdominal aorta, distal to the renal arteries, to the external iliac arteries, although the left renal artery showed reduced flow without complete occlusion.

The intervention successfully restored blood flow to the lower extremities. Post-procedure ABI was 1.11 on the right and 1.03 on the left. Serum CK peaked at 33,559 U/L the day after the procedure, consistent with ischemia-reperfusion injury. Follow-up CT confirmed appropriate stent position and sustained perfusion to all major visceral arteries—except the left renal artery—and to both external and internal iliac arteries (Figures 2a,b). After the procedure, the patient was treated with three antihypertensive agents—a β-blocker, a calcium channel blocker, and an angiotensin receptor blocker—and a proton pump inhibitor. The pulmonary embolism resolved, and no evidence of deep vein thrombosis or in-stent thrombosis was observed. Anticoagulation therapy was deferred with close surveillance. This management decision was individualized for this case and should not be generalized. The pulmonary embolism was considered to be associated with activation of the coagulation–fibrinolytic system secondary to acute aortic dissection rather than deep vein thrombosis. Therefore, the indication for long-term anticoagulation was judged to require careful consideration after the chronic phase.

**Figure 3:**
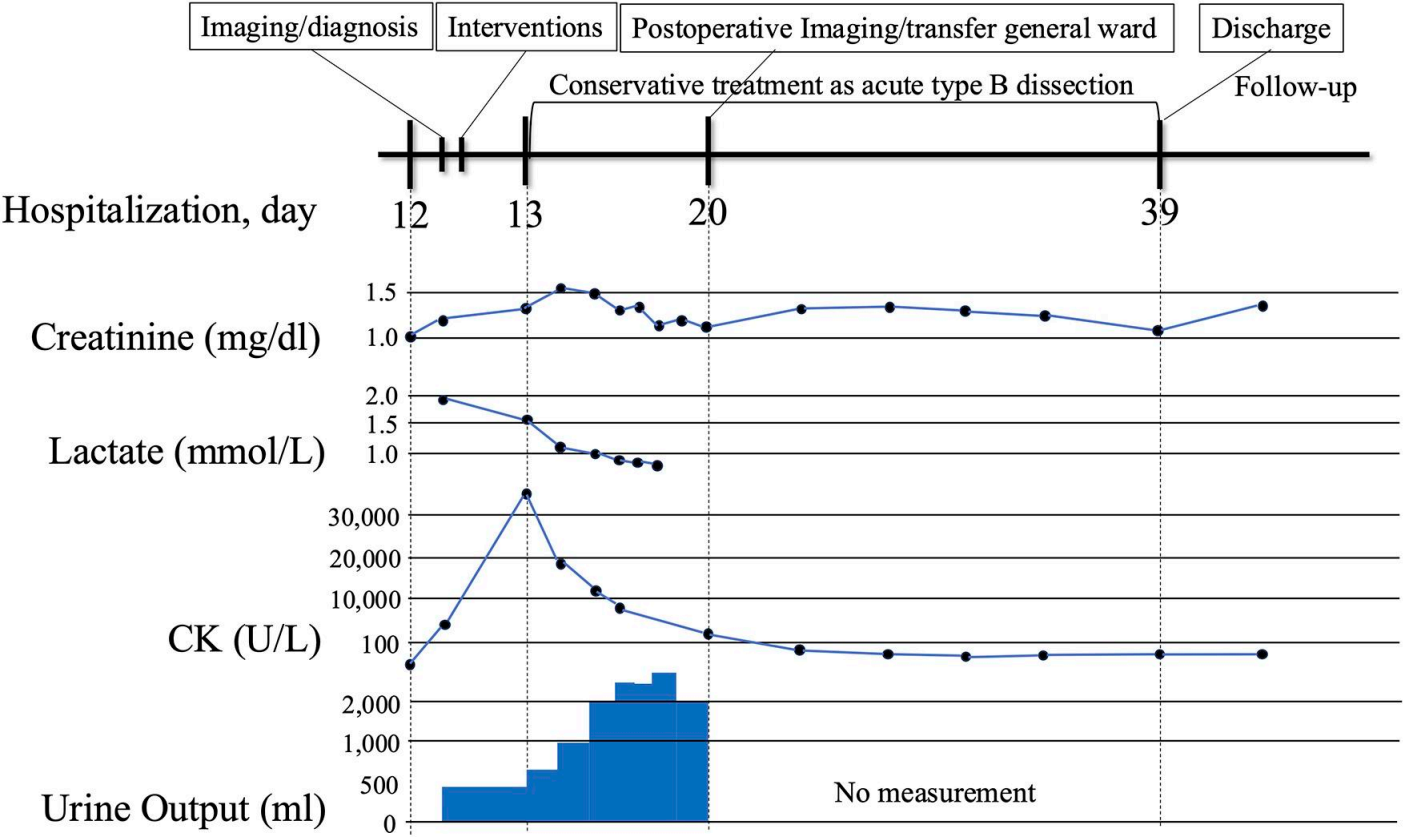
Clinical timeline from symptom onset to follow-up. The timeline begins with symptom onset of acute bilateral lower limb ischemia with relevant laboratory abnormalities (e.g., lactate, creatine kinase, creatinine, urine output), emergency deployment of bridging bare-metal stents, restoration of limb perfusion, and follow-up.

The patient was discharged without complications. Renal function gradually improved postoperatively (Figure 3). Daily measurements confirmed stable bilateral ankle-brachial indices with no evidence of recurrent ischemia. Pre-discharge CTA demonstrated patent visceral branches. Follow-up CT angiographies are scheduled at 3, 6, and 12 months. The 3-month scan showed expansion of the true lumen and a partially thrombosed but still perfused false lumen, with preserved organ perfusion. Blood pressure has been strictly controlled below 130 mmHg systolic. Because there have been no signs of aneurysmal changes or other aortic adverse events, staged TEVAR is not currently planned. Regular surveillance will be continued, and intervention will be considered if future aortic enlargement or other aorta-related complications are detected. At 3 months after discharge, serum creatinine was 1.29 mg/dL, eGFR was 32.5 mL/min/1.73 m^2^, and the ankle-brachial index was 1.05 on the right and 1.06 on the left, indicating good renal function and limb perfusion without any symptoms.

## Discussion

The optimal treatment strategy for acute TBAD complicated by visceral ischemia remains case-dependent, as no standardized regimen has been established. In particular, patients with visceral or lower limb malperfusion face increased rates of mortality and postoperative complications, even following successful aortic replacement, due to ischemia–reperfusion injury in previously underperfused organs and the systemic effects of accumulated metabolic byproducts (3). In the setting of organ malperfusion, endovascular intervention is currently considered the first-line treatment, with thoracic endovascular aortic repair (TEVAR) specifically recommended (1, 4). Extra-anatomic bypass from the axillary artery to the bilateral femoral arteries is another emergency option, particularly in cases of lower extremity malperfusion. However, when ischemia extends to the abdominal organs, the efficacy of this approach becomes uncertain. Consequently, it is generally not regarded as the first-line treatment, with reported mortality and morbidity rates of 5% and 31.6%, respectively (5). While such emergency life-saving interventions are selected based on the patient's clinical condition, even technically successful procedures are associated with a reported mortality rate of 14%, indicating the need for further therapeutic refinement (5). The bridging bare-metal stent technique provides rapid, resource-constrained restoration of multilevel dynamic flow in acute malperfusion while preserving the option for staged, definitive repair (e.g., TEVAR). This approach is particularly valuable in emergent situations or in institutions without immediate access to covered stent grafts (Table 1).

**Table 1:** Comparison of endovascular strategies for complicated or malperfused type B aortic dissection.

Strategy	Typical indications	Device availability	Procedural speed	Limb/Organ perfusion restoration	Need for re-intervention	Key features/Notes
Bridging bare-metal stent (current study)	Rapid stabilization in malperfusion or dynamic obstruction; resource-limited settings	Widely available (no custom device required; availability depends on local inventory and region) Device used: Epic™ self-expanding nitinol stent (Boston Scientific, USA)	Very fast (door-to-reperfusion ≤30 min)	Immediate multilevel flow restoration	Unknown; the report is at the case report level^a^	Preserves options for later definitive repair; suitable for emergency or unstable patients. Potential risks include SINE, subintimal wire passage, and later device apposition challenges during staged TEVAR; these were mitigated by IVUS guidance and avoidance of post-dilatation.
TEVAR-first	Standard of care for complicated TBAD with proximal entry tear	Requires covered stent graft; may be limited by landing zone	Moderate (typically 60–120 min)	Restores true lumen flow mainly proximally	Low–moderate (∼10% at 1 year)^b^	Effective for proximal tears but may not resolve distal malperfusion without adjuncts.
Catheter fenestration	Isolated dynamic obstruction, especially distal or branch-level malperfusion	Widely available (availability may vary by region)	Moderate–fast (45–90 min)	Selective decompression of false lumen	Variable; may require adjunct TEVAR (up to 30%)^c^	Simple and flexible but; suitable for focal distal/branch-level obstruction, limited for extensive dissection
PETTICOAT (Provisional extension to induce complete attachment)	Residual distal collapse after proximal TEVAR	Requires covered + bare stents	Moderate (90–120 min)	Improves distal true lumen expansion	Moderate(∼20% to 30% at 1 year)^d^	Hybrid approach; promotes remodeling but device-intensive
STABLE/STABILISE	Extensive dissection requiring full aortic remodeling	Specialized devices (e.g., composite stent systems)	Slower (120–180 min)	Restores both flow and promotes remodeling	Low–moderate (∼10% to 20% at 1 year)^e^	High device demand; suitable for centers with full endovascular capability

Procedural times are approximate and may vary depending on operator experience, institutional workflow, and device availability.

a Anastacia P et al., J Vasc Surg 2021;74:1143–51.

b Nienaber CA et al., Circulation 2009;120:2519–2528 (INSTEAD-XL trial, 1-year outcomes).

c Jakob H et al., Eur J Cardiothorac Surg 2017;51:329–338. (Variable reintervention rates depending on lesion extent.)

d Lombardi JV et al., J Vasc Surg 2014;59:1544–54 (STABLE trial, 2-year data).

e Lombardi JV et al., J Vasc Surg 2019;70:1072–1081; Nana P., Rev Cardiovasc Med 2023;24(2):34; Faure EM et al., J Thorac Cardiovasc Surg 2021;162:1467–73 (follow-up 6–24 months).

In this case, the patient developed in-hospital malperfusion distal to the abdominal aorta on a Sunday, and it took three hours to obtain a suitable stent graft. Given the severity of pain and the potential for fatal outcome with treatment delay, an emergency endovascular intervention using a self-expanding nitinol stent available onsite was selected as the initial life-saving strategy. This approach preserved blood flow to key branch vessels, including the inferior mesenteric and internal iliac arteries. As a contingency, axillary–femoral artery bypass was prepared and could have been performed immediately if endovascular treatment failed.

Pulmonary embolism in patients with aortic dissection is rare but may result from a hypercoagulable state or embolization from a partially thrombosed false lumen. Previous reports have shown that dynamic flow changes or recanalization can lead to thrombus fragmentation and distal embolization from the false lumen (6, 7). In our case, the occurrence of pulmonary embolism with aortic dissection may reflect a systemic prothrombotic state, potentially mediated by circulating procoagulant factors released from the pre-existing false lumen thrombosis. The mechanism of false lumen reopening is multifactorial, involving persistent re-entry or partial thrombosis with recanalization (8). Although anticoagulation may increase false lumen patency after aortic repair, it has not been consistently associated with poor outcomes (9, 10). Therefore, when clinically indicated, anticoagulation can be used with careful imaging follow-up.

## Data Availability

The original contributions presented in the study are included in the article/Supplementary Material, further inquiries can be directed to the corresponding author.
